# Hemiface Differences in Visual Exploration Patterns When Judging the Authenticity of Facial Expressions

**DOI:** 10.3389/fpsyg.2017.02332

**Published:** 2018-01-10

**Authors:** Yuri Busin, Katerina Lukasova, Manish K. Asthana, Elizeu C. Macedo

**Affiliations:** ^1^Social and Cognitive Neuroscience Laboratory and Developmental Disorders Program, Center for Health and Biological Sciences, Mackenzie Presbyterian University, São Paulo, Brazil; ^2^Center of Mathematics, Computation and Cognition, Federal University of ABC (UFABC), São Bernardo, Brazil; ^3^Department of Humanities and Social Sciences, Indian Institute of Technology Kanpur, Kanpur, India

**Keywords:** emotion judgment, dynamic emotions, eye movements, left side preference, genuine emotions, event-elicited masked emotions, gaze pattern

## Abstract

Past studies have found asymmetry biases in human emotion recognition. The left side bias refers to preferential looking at the left-hemiface when actively exploring face images. However, these studies have been mainly conducted with static and frontally oriented stimuli, whereas real-life emotion recognition takes place on dynamic faces viewed from different angles. The aim of this study was to assess the judgment of genuine vs. masked expressions in dynamic movie clips of faces rotated to the right or left side. Forty-eight participants judged the expressions on faces displaying genuine or masked happy, sad, and fearful emotions. The head of the actor was either rotated to the left by a 45° angle, thus showing the left side of the face (standard orientation), or inverted, with the same face shown from the right side perspective. The eye movements were registered by the eye tracker and the data were analyzed for the inverse efficiency score (IES), the number of fixations, gaze time on the whole face and in the regions of interest. Results showed shorter IESs and gaze times for happy compared to sad and fearful emotions, but no difference was found for these variables between sad and fearful emotions. The left side preference was evident from comparisons of the number of fixations. Standard stimuli received a higher number of fixations than inverted ones. However, gaze time was long on inverted compared to standard faces. Number of fixations on exposed hemiface interacted with the emotions decreasing from happy to sad and fearful. An opposite pattern was found for the occluded hemiface. These results suggest a change in fixation patterns in the rotated faces that may be beneficial for the judgments of expressions. Furthermore, this study replicated the effects of the judgment of genuine and masked emotions using dynamic faces.

## Introduction

Facial expressions allow the exchange of information about affective states and play a crucial role in social cognition of humans. It has been suggested that human face processing is enhanced by a left gaze bias defined by preferential and longer viewing of the left hemiface (the right side of the viewed face; Gilbert and Bakan, 1973; Sackeim et al., 1978; Heller and Levy, 1981; Hisao and Cottrel, 2008). The left side bias was found in children over 5 years of age, but was reduced in 11-year-olds with autism (Chiang et al., 2000; Taylor et al., 2012), which may indicate links with the development of social recognition and interaction. In addition, preferential left side gaze, particularly when unrelated to faces was found also in human 6-month old babies and rhesus monkeys, which may suggest even broader adaptive significance (Guo et al., 2009).

Assessment of the hemifacial asymmetries in emotional expressions showed that the left side is more emotionally expressive and the left-sided facial movements are more pronounced for negative than positive emotions (Borod et al., 1988; Nicholls et al., 2004). Indeed measuring facial muscle movement during emotional expression demonstrated increased movement of the left in comparison with the right hemiface (Dimberg and Petterson, 2000). These findings are in line with studies using composite photographs, created by mirror-reversed images of left–left and/or right–right hemiface, showing that the left composite of faces are judged as more emotionally expressive than the right one (Moreno et al., 1990). Also for posed smiles, produced by actors in the absence of the real emotion stimuli, the left–left composite photographs were judged as more trustworthy than the right ones (Okubo et al., 2013).

To determine which facial features are selected in visual search for more detailed examination, gaze fixation has been examined during judgment of different emotions. In facial expressions of 2D images people fixate their eyes mainly on the eyes and nose region, followed by the mouth and cheeks (Kret et al., 2013; Miellet et al., 2013). However, these regions seem to contribute differently to the recognition depending on the type of emotion being processed. Happy expressions can be recognized after exposure as brief as 20–40 ms, and the most fixated facial region is the mouth, while other regions make little contribution to this recognition (Nusseck et al., 2008; Calvo and Nummenmaa, 2009; Du and Martinez, 2013). Longer exposure times of approximately 100–250 ms are needed for recognition of sad and fearful expressions (Eisenbarth and Alpers, 2011; Du and Martinez, 2013). For recognition of sadness, mainly the eyes, eyebrows, and mouth are looked at Nusseck et al. (2008), Eisenbarth and Alpers (2011). For fear recognition, people mainly fixate the eyes, and the nose region can provide additional information (Schurgin et al., 2014). Interestingly, visual processing of facial regions correlated with the total number of left hemiface fixations and when the eye movements were reduced by short stimuli presentation time, the left side bias was evident (Butler et al., 2005; Butler and Harvey, 2006).

Much of this research has used static faces, which do not closely reflect a natural social interaction. Therefore, a dynamic presentation should provide a more similar representation of the natural environment, as well as more visual cues for local and global feature processing when compared to the use of static presentations (Atkinson et al., 2004; Krumhuber and Manstead, 2009; McLellan et al., 2010; Harris et al., 2014). In the case of basic expressions, there is a consensus over a stereotypical pattern of facial activation that can be adequately perceived and recognized as one emotion (Nusseck et al., 2008; Cristinzio et al., 2010). This pattern strongly depends on deformation of distinct morphological facial areas [action units (AUs); Ekman and Friesen, 1978]. For example, happy emotions can be produced by AU such as crow’s feet wrinkles around the eyes together with pulling up of the lip corners, known as the Duchenne marker (D) (Ekman and Rosenberg, 1997). This marker is produced by the contraction of the orbiculares oculi and zygomaticus major muscles and is thought to be a sign of a genuine smile in static emotional faces (Peron and Roy-Charland, 2013). A study that examined the importance of the D marker in discrimination between spontaneous and deliberate smiles in static and dynamic displays by healthy adults showed that the marker was not the most stable cue for rating smiles and the selection of preferable visual features follows a different pattern (Krumhuber and Manstead, 2009). The importance of dynamic expressions, such as movie clips, lies in the possibility of seeing the onset, apex, and offset phases of the expressed emotion, thus increasing perceptual sensitivity (Krumhuber and Kappas, 2005). Furthermore, it seems that both the features and the event’s timing play an important role in facial perception and emotional recognition. The observer may ignore the AU markers of negative emotion in the eye regions when there is a smiling mouth. This effect tended to be bigger if the mouth motion came only after a change in the eyes (Iwasaki and Noguchi, 2016).

Thus the evidence shows that the perception of timing in facial movement enhances the facial expression recognition (Atkinson et al., 2004; Harris et al., 2014; Weyers et al., 2016; Yan et al., 2017). However, not many studies investigated how the left side bias is affected in these dynamic presentations, and the influence of timing. In one study that investigated this question, a stronger left hemiface bias was found in dynamic displays compared to static faces or face-like objects. The preference to explore the right side of the face was most evident in the eye region and it was present even in the mirrored face stimuli (Everdell et al., 2007).

The current study aimed to investigate: (i) the pattern of gaze on rotated dynamic human faces showing three basic human emotions (happy, sad, and fearful), and (ii) the effect of left side bias, showing the same clip from the left (standard) and right (inverted) side in a 45° angle. We hypothesized that recognizing happy emotion in movie clips requires less visual processing, an effect previously reported only in static images (Nusseck et al., 2008; Korb et al., 2014). On the other hand, inverted images pose higher demands on visual processing since they offer a non-preferential side of the human face; thus, we expect to find the left side preference for visual perception (Chelnokova and Laeng, 2011). Additional difficulty is expected when discriminating between genuine and masked expressions due to temporal incongruence and asymmetry of AU markers, since studies indicate that in dynamic faces, the typical AU marker’s deformation may be overridden by other temporal cues (Krumhuber and Kappas, 2005).

## Materials and Methods

### Participants

A total of 47 undergraduate students of the Mackenzie Presbyterian University volunteered for the experiment. This sample size is consistent with many other studies on this subject (Chelnokova and Laeng, 2011; Du and Martinez, 2013). All volunteers had normal or corrected-to-normal vision. Participants with a history of head surgery, head trauma or seizures, drug addiction, psychosis, or dementia were excluded. One participant was later excluded from the experiment due to insufficient eye-tracking data. Thus, 46 participants (*M* = 22.65 years old, *SD* = 3.22) were included in the analyses. Female (*N* = 30) and male participants did not differ with respect to age and handedness (*p* > 0.05). This study was carried out in accordance with the recommendations of Mackenzie Presbiterian University Ethics committee, that reviewed and approved the project. The study was approved by the local ethics committee (CAAE No. 50307815.8.0000.0084) and each participant provided written informed consent prior to the experiment.

### Stimuli

Movie clips were selected from the Computerized Test of Primary Emotion Perception (Miguel and Primi, 2014). The test shows genuine and event-elicited masked facial expressions for a variety of human emotions. Each clip depicted the head and the upper part of the shoulders of a person expressing an emotion, with the head rotated horizontally 45° to the left side. Each clip was of 4 s duration.

Miguel and Primi (2014) recorded videos of individuals viewing pictures of different emotional content from the International Affective Pictures System (IAPS) in order to produce genuine emotional expressions. The incongruent emotion videos were produced when individuals had to mask the genuine expressions elicited by the picture with one out of eight primary emotions. For example, when viewing a happy picture, the individual in the video could produce either a sad or another facial expression. These emotions were labeled as event-elicited masked emotions. The videos were administered to 310 naïve participants who judged the videos for the type and veracity of the expressed emotion (Primi, 2014).

For the purpose of this study, only three basic emotions were chosen: happy, sad, and fearful expressions. The emotions were presented by 12 different actors (three men and nine women) and there were four actors per emotion. Each actor performed both genuine and masked expressions. The clips were matched on other physical properties of the image such as the background color, luminosity, and the size and position of the face in the background. Each clip was recorded showing the left side of the face from a 45° angle (labeled as standard) and was mirrored to show the actors from a right-hand 45° angle (labeled as inverted). Each clip was presented four times in pseudo-randomized sequences in two runs separated by a 5 min rest period. In total, the participants judged 96 clips (48 in each run): 24 standard movie clips for genuine emotions (i.e., happy, sad, and fearful), 24 standard movie clips for masked emotions, 24 inverted movie clips for genuine emotions, and 24 inverted movie clips for masked emotions. In each group with 24 clips, the same number of movies showed happy, sad, and fearful emotions, eight of each. Busin (2011, Unpublished) validated all the clips with healthy participants in a pilot study. In this study (*N* = 13) genuine displays were correctly rated as genuine (*M* = 70%, *SD* = 4.6) and masked (*M* = 43%, *SD* = 4.9). Also, all emotional expressions were recognized accordingly, including happy (*M* = 80%, *SD* = 4), sad (*M* = 80%, *SD* = 3.9), and fearful (*M* = 10%, *SD* = 3).

### Eye Tracking and Measures

Using the Eye Gaze Edge 1750 eye tracker (LC Technologies, Inc., United States) the current study collected position information related to both eyes. The eye tracking data analysis program NYAN was used for off-line data processing. The default settings for fixation detection considered parameters of gaze deviation from a threshold of 25 pixels for the minimum of six samples, with a recording frequency of 120 Hz. The movie clips were presented on a 19-inch flat screen color monitor (1490 × 900 pixels) at a viewing distance of 60 cm. In addition, the eye position was monitored in real-time by the experimenters on a second monitor used both for instruction and quality check.

### Procedure

At the beginning of the experiment, all participants were given detailed instructions and a brief training. The participants were instructed to watch the movie clips and decide whether the presented emotion was genuine or masked. After each movie clip, a black screen with a fixation cross appeared, during which the participant was instructed to respond to the clip by pressing one of two keys on the keyboard: “v” for genuine, “m” for masked. Once the response was given, a new movie clip was presented. All tests were conducted in the same room with the lights off, without sounds, and in the presence of an experimenter.

### Data Analysis

All statistical data analyses were performed using the IBM SPSS 20.0 program. For eye-tracking data, we performed conventional analysis of variance (ANOVA) with emotion (happy, sad, fearful), veracity (genuine, masked) and side (standard, inverted) as within-subject factors. Based on previous research findings, three basic dependent measures were considered: (1) *inverse efficiency score (IES)*: computed for each participant’s average response time divided by the total of correct responses in order to account for any possibilities of speed-accuracy trade-offs (Townsend and Ashby, 1983); (2) *number of fixations*: the average number of eye fixations in the whole movie clip; and (3) *gaze duration*: the average duration of all fixations in the whole movie clip.

## Results

### Inverse Efficiency Score (IES)

Using *IES* scores as the dependent variable, a three-way ANOVA was conducted. Results revealed a significant main effect for veracity (*F*_(1,45)_ = 6.96, *p* = 0.01, ${\text{n}}_{\text{G}}^{2}$ = 0.023) and emotion (*F*_(2,90)_ = 4.75, *p* = 0.01, ${\text{n}}_{\text{G}}^{2}$ = 0.021). The *post hoc* Bonferroni comparison indicated lower *IES* for happy (*M* = 194 ms) than sad (*M* = 239 ms) but there was not difference for sad and fearful (*M* = 243 ms) emotions. A lower *IES* was found for genuine (*M* = 202 ms) compared to masked (*M* = 249 ms) emotions.

### Number of Fixations

Results of a three-way ANOVA examining the number of fixations revealed statistically significant main effects for veracity (*F*_(1,45)_ = 4.62, *p* = 0.04, ${\text{n}}_{\text{G}}^{2}$ = 0.002) and side (*F*_(1,45)_ = 16.48, *p* < 0.001, ${\text{n}}_{\text{G}}^{2}$ = 0.007), but not for emotion. More fixations were made on the genuine (*M* = 8.87) compared to masked (*M* = 8.61) expressions and on standard (*M* = 9.01) compared to inverted (*M* = 8.47) faces.

### Gaze Duration

A three-way ANOVA showed significant main effects for side (*F*_(1,45)_ = 4.18, *p* < 0.05, ${\text{n}}_{\text{G}}^{2}$ = 0.004) and emotion (*F*_(2,90)_ = 5.36, *p* < 0.01, ${\text{n}}_{\text{G}}^{2}$ = 0.005). The *post hoc* Bonferroni comparison indicated shorter gaze duration on happy (*M* = 403 ms) than fearful (*M* = 422 ms) emotions, but no difference was found for fearful and sad (*M* = 428 ms). Gaze was longer on the inverted (*M* = 429 ms) compared to standard (*M* = 406 ms) faces (Supplementary Material 1).

### Analyses of ROI

**FIGURE 1:**
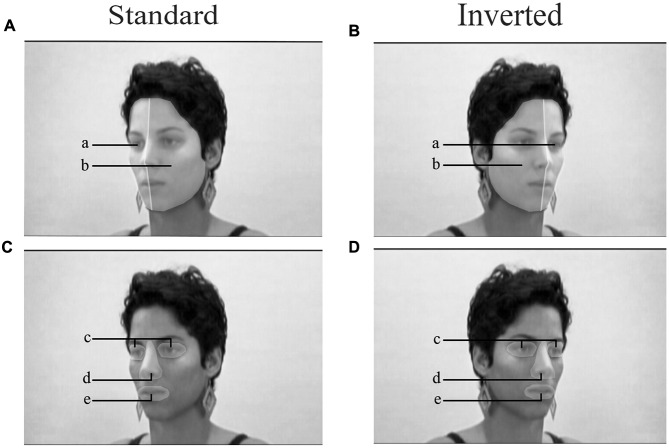
The regions of interest (ROIs). Faces in standard orientation **(A,C)** and inverted **(B,D)** with ROIs defined as occluded hemi-face (a), exposed hemi-face (b), eyes region (c), nose region (d), mouth region (e). The variables of interest were extracted from ROIs and the three-way ANOVA was used with emotion as a between-groups factor.

To better characterize the visual exploration pattern, the number of fixations and gaze time on regions of interest (ROI) was computed (**Figure 1**). ROIs were selected as follows: exposed half-face and occluded half-face (**Figures 1A,B**; ROI a, b). The aim was to show the pattern of visual exploration of the face as a function of veracity and side. The three-way ANOVA was performed for each emotion with veracity (genuine, masked) and side (standard, inverted) as within-subject factors.

**FIGURE 2:**
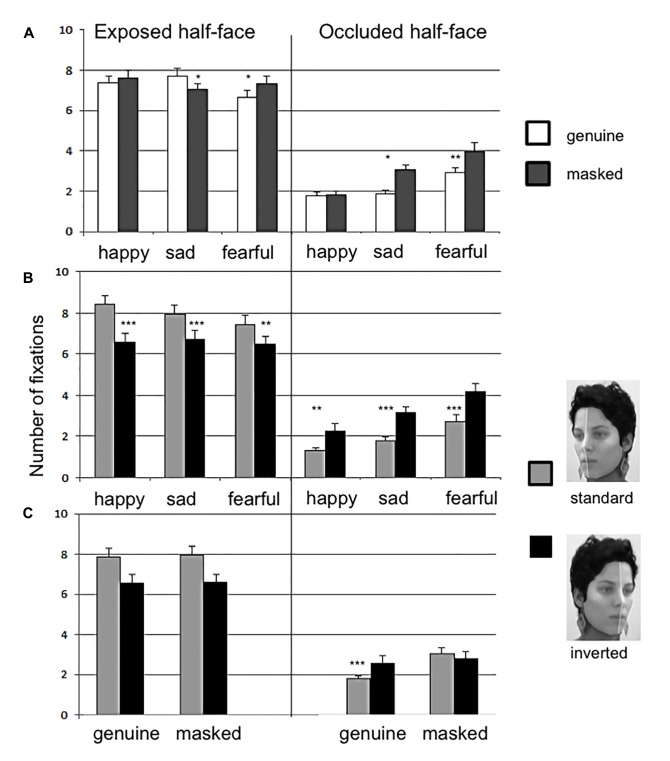
The number of fixations on the ROIs. Exposed half-face (left side) and occluded half-face (right side) graphs show distribution of fixations in interaction with emotion and veracity **(A)**; emotion and side **(B)**; and veracity and side **(C)**. The error bars show standard error and statistical significance is marked by ^∗^*p* < 0.5; ^∗∗^*p* < 0.01; ^∗∗∗^*p* < 0.001.

For the number of fixations on the exposed half-faces, the main effects were found for side (*F*_(1,45)_ = 12.85, *p* < 0.001, ${\text{n}}_{\text{G}}^{2}$ = 0.053) and emotion (*F*_(2,90)_ = 9.79, *p* < 0.001, ${\text{n}}_{\text{G}}^{2}$ = 0.007). Furthermore, there were interactions between emotion and veracity (*F*_(2,90)_ = 25.75, *p* < 0.001, ${\text{n}}_{\text{G}}^{2}$ = 0.013) and emotion and side (*F*_(2,90)_ = 7.55, *p* < 0.01, ${\text{n}}_{\text{G}}^{2}$ = 0.005), but not veracity and side. The standard oriented faces received more fixations than inverted faces in all the emotions and the interaction is depicted in **Figure 2**.

For the number of fixations on occluded half-face ROI, the main effects were found for veracity (*F*_(1,45)_ = 32.74, *p* < 0.001, ${\text{n}}_{\text{G}}^{2}$ = 0.04) and emotion (*F*_(2,90)_ = 38.31, *p* < 0.001, ${\text{n}}_{\text{G}}^{2}$ = 0.116). There were interactions between emotion and veracity (*F*_(2,90)_ = 7.37, *p* < 0.01, ${\text{n}}_{\text{G}}^{2}$ = 0.023), emotion and side (*F*_(2,90)_ = 39.85, *p* < 0.001, ${\text{n}}_{\text{G}}^{2}$ = 0.121) and veracity and side (*F*_(2,90)_ = 12.32, *p* < 0.001, ${\text{n}}_{\text{G}}^{2}$ = 0.024). The direction of the interactions is depicted in **Figure 2**.

The gaze duration on the exposed half-faces showed a significant main effect for emotion (*F*_(2,90)_ = 6.30, *p* < 0.01, ${\text{n}}_{\text{G}}^{2}$ = 0.016). The exposed half-faces of happy emotions (*M* = 388 ms) received significantly shorter gaze than sad (*M* = 423 ms, *p* < 0.05), and fearful (*M* = 429 ms) emotions. There was no difference in gaze between sad and fearful (Bonferroni correction). No main effect was found for the gaze duration on occluded half-face ROI.

**FIGURE 3:**
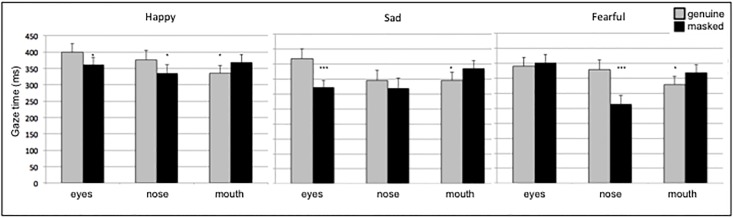
The gaze time on eyes, nose and mouth ROIs. The gaze time duration as a function of region, emotion, and veracity. The error bars show standard error and statistical significance is marked by ^∗^*p* < 0.5; ^∗∗^*p* < 0.01; ^∗∗∗^*p* < 0.001.

For the eyes, nose, and mouth ROI, ANOVA (**Figures 1C,D**; ROI c, d, e) was performed on gaze time with emotion (happy, sad, fearful), facial region (eye, nose, mouth), veracity (genuine, masked), and side (standard, inverted) as within-subject factors. The significant two-way interaction were found for emotion and region (*F*_(4,176)_ = 9.64, *p* < 0.001, ${\text{n}}_{\text{G}}^{2}$ = 0.022) and region and veracity (*F*_(2,88)_ = 11.21, *p* < 0.001, ${\text{n}}_{\text{G}}^{2}$ = 0.025). There was a three-way interaction of region, emotion and veracity (*F*_(4,176)_ = 6.60, *p* < 0.001, ${\text{n}}_{\text{G}}^{2}$ = 0.016). Pairwise comparison indicated longer gaze time on nose and eyes region in genuine happy emotions; longer gaze on eyes in genuine sad; and on nose in genuine fearful emotions. Longer gaze time was found for the mouth region in all masked emotions. The results are depicted in **Figure 3**.

## Discussion

The present study revealed that the pattern of gaze on dynamic human faces of three basic human emotions varied according to the side of the rotated face and the type of emotion being judged. Faces exposed from the left side had more fixations and the number of fixations decreased progressively from happy to sad and then fearful emotions. This pattern was evident mainly in the exposed hemiface, which suggests that subjects directed their gaze toward most salient features of the face. The occluded hemiface was fixated to a smaller extent and a different pattern was found; the number of fixations increased from happy to sad and fearful emotions. Thus, subjects may develop flexible scanning routines in order to gather additional information when facing rotated dynamic human faces. In this case, fixating in occluded facial regions seems to be associated with the increasing difficulty to judge the veracity of the presented emotion. A smaller number of fixations on the exposed right hemiface could evidence more efficient visual processing. However, when we look at the occluded right hemiface, the increase in the number of fixations indicates that there is much more need for additional visual clue than in the left occluded hemiface. These results evidence the presence of an asymmetry bias in dynamic emotions and indicate a specific strategy to extract additional visual clues for correct emotional judgment.

Previous studies showed that the left side of the face is more active than the right side when we express emotions. In addition, the aesthetic feeling is generally better for the left-faced images (Chiang et al., 2000; Adolphs, 2002; Okubo et al., 2013). People more often show the left cheek when they take selfies (Lindell, 2017) and portraits of faces are depicted mainly from the left side (McManus and Humphrey, 1973). Blackburn and Schirillo (2012) investigated preferences for the recognition of emotions according to the face’s orientation. They recorded the reaction time and judgment of pleasantness of photos with smiling expressions rotated horizontally by 15°, emphasizing either the left or right side comparisons. The results indicated a left side bias, since it was more pleasurable to look at pictures in which the left side was more apparent, and the recognition time was lower in this condition. The pattern of visual exploration found in the present study is aligned with these findings. However, it is not clear whether this asymmetric bias may be supported by neuro-functional maturation when it comes to the face perception (Chiang et al., 2000; Adolphs, 2002) or is rather a culturally defined viewing preference (Marzoli et al., 2014).

Genuine and masked emotions are characterized by different brain states during their production, since the actor who was asked to produce a happy face was viewing a sad scene. Studies suggest that this incongruence is expected to produce asymmetry in the dynamics of emotion expression, by irregular onset/offset time of the muscle deformation, for example in a fake smile. Iwasaki and Noguchi (2016) showed that the change in mouth movements impaired the emotion perception of micro-expression in the eye regions, but only when showed after and not before the eye change. The diagnostic information for the emotional expression may be concentrated in different regions of the static face (Ekman and Friesen, 1978; Nusseck et al., 2008; Cristinzio et al., 2010). In a dynamic display of rotated faces, length of gaze on preferential facial regions varied as a function of the type of emotion. For genuine sad emotions the eyes were preferred, while for fear the nose was preferentially gazed. The increase in gaze time on mouth region in all masked emotions may be explained by increased difficulty in judging emotion’s veracity. Buchan et al. (2007) showed that even modest increases in difficulty alter gaze patterns.

The results of this study showed longer IES in judging sad and fearful expressions compared to the happy expressions, combining that with shorter gaze time on happy faces, it indicates the effect known as happy emotion facilitation. This is in line with other studies of emotions in static faces, which defend that some expressions, such as a smile, are readily recognized due to deformation of muscles in only one or two facial regions (Nusseck et al., 2008; Du and Martinez, 2013). The genuine smile in static emotional faces is judged by the presence of crow’s feet wrinkles around the eyes known as the Duchenne marker (Peron and Roy-Charland, 2013). However, as shown by studies with dynamic presentation of emotion, the temporal development of the expressions that change gradually over time produce subtle cues that enhance the perception of embedded information. These additional cues such as mouth deforming and opening reduce the importance of the eye region typically found is static face stimuli (Krumhuber and Manstead, 2009; Krumhuber et al., 2013; Korb et al., 2014). When looking at dynamic emotions, average gaze time was the longest for the nose region of happy faces, the mouth of sad faces, and the eyes of fearful faces. Considering that this pattern was influenced by the genuine/masked factor, it is plausible that these results indicate a goal-driven viewing strategy.

Some limitations of this study must be acknowledged. First, our sample was limited in diversity (i.e., more than half were psychology undergraduate students). Second, all the movie clips were presented at the center of the screen, and the only manipulation was the mirroring of the faces. Thus, the extrapolation of conclusions on hemifield perception should be careful, since this variable was not controlled in our study. Finally, we also make no claim whether the perception of genuine and masked emotions behaves in a similar fashion for emotions other than happiness, sadness, and fear. Further studies should attend to these questions.

In summary, this study provides insight into the hemiface differences in emotion judgment and evidence of the asymmetry bias in dynamic stimuli contributing to understanding basic processes of social interactions.

## Author Contributions

YB and EM developed and contributed to the study design. YB and MA performed the data collection. KL performed the data analysis. All authors contributed to data interpretation and writing the manuscript, and approved the final version of the manuscript for submission.

## Conflict of Interest Statement

The authors declare that the research was conducted in the absence of any commercial or financial relationships that could be construed as a potential conflict of interest.

## References

[B1] Adolphs R. (2002). Neural systems for recognizing emotion. *Curr. Opin. Neurobiol.* 12 169–177. 10.1016/S0959-4388(02)00301-X 12015233

[B2] Atkinson A. P. Dittrich W. H. Gemmell A. J. Young A. W. (2004). Emotion perception from dynamic and static body expressions in point-light and full-light displays. *Perception* 33 717–746. 10.1068/p5096 15330366

[B3] Blackburn K. Schirillo J. (2012). Emotive hemispheric differences measured in real-life portraits using pupil diameter and subjective aesthetic preferences. *Exp. Brain Res.* 219 447–455. 10.1007/s00221-012-3091-y 22526951

[B4] Borod J. C. Kent J. Koff E. Martin C. Alpert M. (1988). Facial asymmetry while posing positive and negative emotions: support for the right hemisphere hypothesis. *Neuropsicologia* 26 759–764. 10.1016/0028-3932(88)90013-9 3211295

[B5] Buchan J. N. Paré H. Munhall K. G. (2007). Spatial statistics of gaze fixations during dynamics face processing. *Soc. Neurosci.* 2 1–13. 10.1080/17470910601043644 18633803

[B6] Butler S. Gilchrist I. D. Burt D. M. Perrett D. I. Jones E. Harvey M. (2005). Are the perceptual biases found in chimeric face processing reflected in eye-movement patterns? *Neuropsychologia* 43 52–59. 10.1016/j.neuropsychologia.2004.06.005 15488905

[B7] Butler S. H. Harvey M. (2006). Perceptual biases in chimeric faces processing: Eye-movement pattern biases in chimeric face processing: Eye-movement patterns cannot explain it all. *Brain Res* 1124 96–99. 10.1016/j.brainres.2006.09.069 17070503

[B8] Calvo M. G. Nummenmaa L. (2009). Eye-movement assessment of the time course in facial expression recognition: neurophysiological implications. *Cogn. Affect. Behav. Neurosci.* 9 398–411. 10.3758/CABN.9.4.398 19897793

[B9] Chelnokova O. Laeng B. (2011). Three-dimensional information in face recognition? an eye-tracking study. *J. Vis.* 11 1–15. 10.1167/11.13.27 22131448

[B10] Chiang C. H. Ballantyne A. O. Trauner D. A. (2000). Development of perceptual asymmetry for free viewing of chimeric stimuli. *Brain Cogn.* 44 415–424. 10.1006/brcg.1999.1202 11104534

[B11] Cristinzio C. N’Diaye K. Seeck M. Vuilleumier P. Sander D. (2010). Integration of gaze direction and facial expression in patients with unilateral amygdala damage. *Brain* 133(Pt 1), 248–261. 10.1093/brain/awp255 19828596

[B12] Dimberg U. Petterson M. (2000). Facial reactions to happy and angry facial expressions: evidence for right hemisphere dominance. *Psychophysiology* 37 693–696. 10.1111/1469-8986.3750693 11037045

[B13] Du S. Martinez A. M. (2013). Wait, are you sad or angry? Large exposure time differences required for the categorization of facial expressions of emotion. *J. Vis.* 13 1–14. 10.1167/13.4.13 23509409 PMC3604912

[B14] Eisenbarth H. Alpers G. W. (2011). Happy mouth and sad eyes: scanning emotional facial expressions. *Emotion* 11 860–865. 10.1037/a0022758 21859204

[B15] Ekman P. Friesen W. V. (1978). *Facial Action Coding System: A Technique for the Measurement of Facial Movement.* Palo Alto, CA: Consulting Psychologists Press.

[B16] Ekman P. Rosenberg E. L. (1997). *What the Face Reveals: Basic and Applied Studies of Spontaneous Expression Using the Facial Action Coding System (FACS).* New York, NY: Oxford University Press.

[B17] Everdell I. T. Marsh H. Yurick M. D. Munhall K. G. Paré M. (2007). Gaze behaviour in audiovisual speech perception: asymmetrical distribution of face-directed fixations. *Perception* 36 1535–1545. 10.1068/p5852 18265836

[B18] Gilbert C. Bakan P. (1973). Visual asymmetry in perception of faces. *Neuropsychologia* 11 355–362. 4792186 10.1016/0028-3932(73)90049-3

[B19] Guo K. Meints K. Hall C. Hall S. Mills D. (2009). Left gaze bias in humans, rhesus monkeys and domestic dogs. *Anim. Cogn.* 12 409–418. 10.1007/s10071-008-0199-3 18925420

[B20] Harris R. J. Young A. W. Andrews T. J. (2014). Dynamic stimuli demonstrate a categorical representation of facial expression in the amygdala. *Neuropsychologia* 56 47–52. 10.1016/j.neuropsychologia.2014.01.005 24447769 PMC3988993

[B21] Heller W. Levy J. (1981). Perception and expression of emotion in right-handers and left-handers. *Neuropsychologia* 19 263–272. 7254505 10.1016/0028-3932(81)90110-x

[B22] Hisao J. H. Cottrel G. (2008). Two fixations suffice in face recognition. *Psychol. Sci.* 19 998–1006. 10.1111/j.1467-9280.2008.02191.x 19000210 PMC7360057

[B23] Iwasaki M. Noguchi Y. (2016). Hiding true emotions: micro-expressions in eyes retrospectively concealed by mouth movements. *Sci. Rep.* 6 1–10. 10.1038/srep22049 26915796 PMC4768101

[B24] Korb S. With S. Niedenthal P. Kaise S. Grandjean D. (2014). The perception and mimicry of facial movements predict judgments of smile authenticity. *PLOS ONE* 9:e99194. 10.1371/journal.pone.0099194 24918939 PMC4053432

[B25] Kret M. E. Stekelenburg J. J. Roelofs K. de Gelder B. (2013). Perception of face and body expressions using electromyography, pupillometry and gaze measures. *Front. Psychol.* 4:28. 10.3389/fpsyg.2013.00028 23403886 PMC3567353

[B26] Krumhuber E. Kappas A. (2005). Moving smiles: the role of dynamic components for the perception of the genuineness of smiles. *J. Nonver. Behav.* 29 3–24. 10.1007/s10919-004-0887-x

[B27] Krumhuber E. G. Kappas A. Manstead A. S. R. (2013). Effects of dynamic aspects of facial expressions: a review. *Emotion Review* 5 41–46. 10.1177/1754073912451349

[B28] Krumhuber E. G. Manstead A. S. R. (2009). Can Duchenne smiles be feigned? New evidence on felt and false smiles. *Emotion* 9 807–820. 10.1037/a0017844 20001124

[B29] Lindell A. (2017). Consistently showing your best side? Intra-individual consistency in selfie pose orientation. *Front. Psychol.* 8:246. 10.3389/fpsyg.2017.00246 28270790 PMC5318447

[B30] Marzoli D. Prete G. Tommasi L. (2014). Perceptual asymmetry and handedness: a neglected link? *Front. Psychol.* 5:163. 10.3389/fpsyg.2014.00163 24592250 PMC3938099

[B31] McLellan T. Johnston L. Dalrymple-Alford J. Porter R. (2010). Sensitivity to genuine versus posed emotion specified in facial displays. *Cogn. Emot.* 24 1277–1292. 10.1080/02699930903306181

[B32] McManus I. C. Humphrey N. (1973). Turning the left cheek. *Nature* 243 271–272. 10.1038/243271a0

[B33] Miellet S. Vizioli L. He L. Zhou X. Caldara R. (2013). Mapping face recognition information use across cultures. *Front. Psychol.* 4:34. 10.3389/fpsyg.2013.00034 23430143 PMC3576804

[B34] Miguel F. K. Primi R. (2014). Creation of emotional expressions videos using multimedia stimuli. *Teoria Prát.* 16 155–168.

[B35] Moreno C. R. Borod J. Welkowitz J. Alpert M. (1990). Lateralization for the perception and expression of facial expression as a function of age. *Neuropsychologia* 28 199–209. 10.1016/0028-3932(90)90101-S 2314574

[B36] Nicholls M. E. R. Ellis B. E. Clement J. G. Yoshino M. (2004). Detecting hemifacial asymmetries in emotional expression with three-dimensional computerized image analysis. *Proc. R. Soc. Lond.* 271 663–668. 10.1098/rspb.2003.2660 15209097 PMC1691649

[B37] Nusseck M. Cunningham D. W. Wallraven C. Bülthoff H. H. (2008). The contribution of different facial regions to the recognition of conversational expressions. *J. Vis.* 8 1–23. 10.1167/8.8.1 18831624

[B38] Okubo M. Ishikawa K. Kobayashi A. (2013). No trust on the left side: hemifacial asymmetries of trustworthiness an emotional expressions. *Brain Cogn.* 82 181–186. 10.1016/j.bandc.2013.04.004 23673250

[B39] Peron M. Roy-Charland A. (2013). Analysis of eye movements in the judgment of enjoyment and non-enjoyment smiles. *Front. Psychol.* 4:659. 10.3389/fpsyg.2013.00659 24069013 PMC3781329

[B40] Primi R. (2014). Psychometric study of the computerized test of primary emotions perception. *Aval. Psicol.* 13 1–9.

[B41] Sackeim H. A. Gur R. C. Saucy M. C. (1978). Emotions are expressed more intensely on the left side of the face. *Science* 202 434–436. 705335 10.1126/science.705335

[B42] Schurgin W. M. Nelson J. Lida S. Ohira H. Chiao J. Y. Franconeri S. L. (2014). Eye movements during emotion recognition in faces. *J. Vis.* 14 14. 10.1167/14.13.14 25406159

[B43] Taylor S. Workman L. Yeomans H. (2012). Abnormal patterns of cerebral lateralization as revealed by the universal chimeric faces task in individuals with autistic disorder. *Laterality* 17 428–437. 10.1080/1357650X.2010.521751 22690895

[B44] Townsend J. T. Ashby F. G. (1983). *The Stochastic Modeling of Elementary Psychological Processes.* Cambridge: Cambridge University Press.

[B45] Weyers P. Mühleberger A. Hefele C. Pauli P. (2016). Electromyographic response to static and dynamic avatar emotional facial expressions. *Psychophysiology* 43 450–453. 10.1111/j.1469-8986.2006.00451.x 16965606

[B46] Yan X. Young A. W. Andrews T. J. (2017). Differences in holistic processing do not explain cultural differences in the recognition of facial expression. *Q. J. Exp. Psychol. (Hove)* 70 2445–2459. 10.1080/17470218.2016.1240816 27766927

