# Supplementary material for: Hemiface Differences in Visual Exploration Patterns When Judging the Authenticity of Facial Expressions
Source: Front Psychol. 2018 Jan 10;8:2332. doi: 10.3389/fpsyg.2017.02332 (PMC5767895; doi:10.3389/fpsyg.2017.02332)
Supplement: Supplementary file 1 [file Data_Sheet_1.docx]

**Supplementary material 1.** Average score and Standard Deviation (SD) for the Inverse Efficiency, Number of fixations and Gaze duration.

| **Genuine** | | | | | | **Masked** | | | | |
| --- | --- | --- | --- | --- | --- | --- | --- | --- | --- | --- |
|  | Happy | Sad | | Fearful |  | Happy | Sad | Fearful |  | |
| **Inverse Efficiency Score** | | | | | | | | | | |
| Standard | 185 (21) | | 211 (36) | 205 (22) |  | 195 (18) | 263 (50) | 264 (36) | |  |
|  |  | |  |  |  |  |  |  | |  |
| Inverted | 196 (22) | | 187 (16) | 226 (22) |  | 198 (18) | 312 (44) | 261 (33) | |  |
| **Number of fixations** | | | | | | | | | | |
| Standard | 8.96(.45) | 9.17 (.50) | | 9.27 (.53) |  | 9.30 (.47) | 8.53 (.41) | 8.85 (.48) |  | |
| Inverted | 8.29 (.46) | 8.76 (.50) | | 8.77 (.49) |  | 8.34 (.48) | 8.32 (.51) | 8.34 (.52) |  | |
| **Gaze duration** | | | | | | | | | | |
| Standard | 392 (21) | 425 (29) | | 397 (29) |  | 393 (21) | 418 (22) | 413 (21) |  | |
| Inverted | 399 (25) | 438 (29) | | 404 (25) |  | 426 (25) | 431 (29) | 474 (28) |  | |
